# Supplementary material for: Irisin Is a Positive Regulator for Ferroptosis in Pancreatic Cancer
Source: Mol Ther Oncolytics. 2020 Aug 5;18:457–66. doi: 10.1016/j.omto.2020.08.002 (PMC7475648; doi:10.1016/j.omto.2020.08.002)
Supplement: Document S1. Figures S1 and S2 and Tables S1 and S2 [file mmc1.pdf]

OMTO, Volume *18*

Supplemental Information

Irisin Is a Positive Regulator for Ferroptosis in Pancreatic Cancer

Bao Chen Yang and Po Sing Leung

Irisin is a positive regulator for ferroptosis in pancreatic cancer

Bao Chen Yang and Po Sing Leung*

Supplementary data

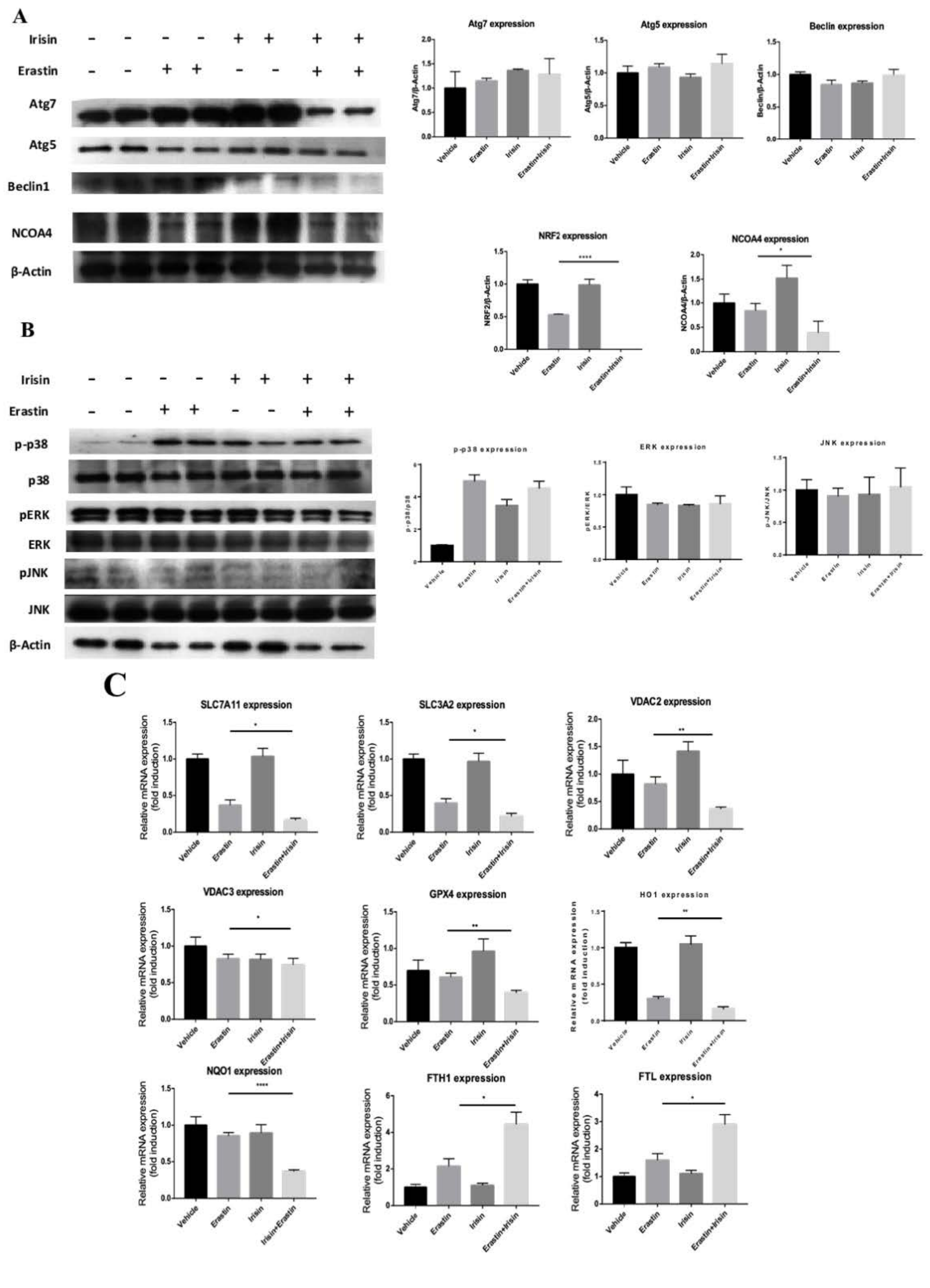

Figure S1. Irisin effects on autophagy-related proteins in ferroptotic PANC-1 cells. (A) Protein expression of Atg7, Atg5, Beclin, and NCOA4 after 12 h treatment with irisin and/or erastin (β-Actin was used as a loading control). (B) No significant effects on p-

p38/p38, pERK/ERK, and pJNK/JNK levels were found. Erastin effects on the transcription of system X_c^- (*SLC7A11*, *SLC3A2*, *GPX4*), ROS metabolism (*VDAC2*, *VDAC3*, *HO1*, *NQO1*), and iron metabolism (*FTL*, *FTH1*) genes were enhanced significantly in the presence of irisin (20 μM erastin, 100 nM irisin, 12 h. treatment period). All data are means ± SEMs, $n \geqslant 3$/group; *p<. .5, **p< .1, ****p < .0001 vs. Erastin group (paired t-tests).

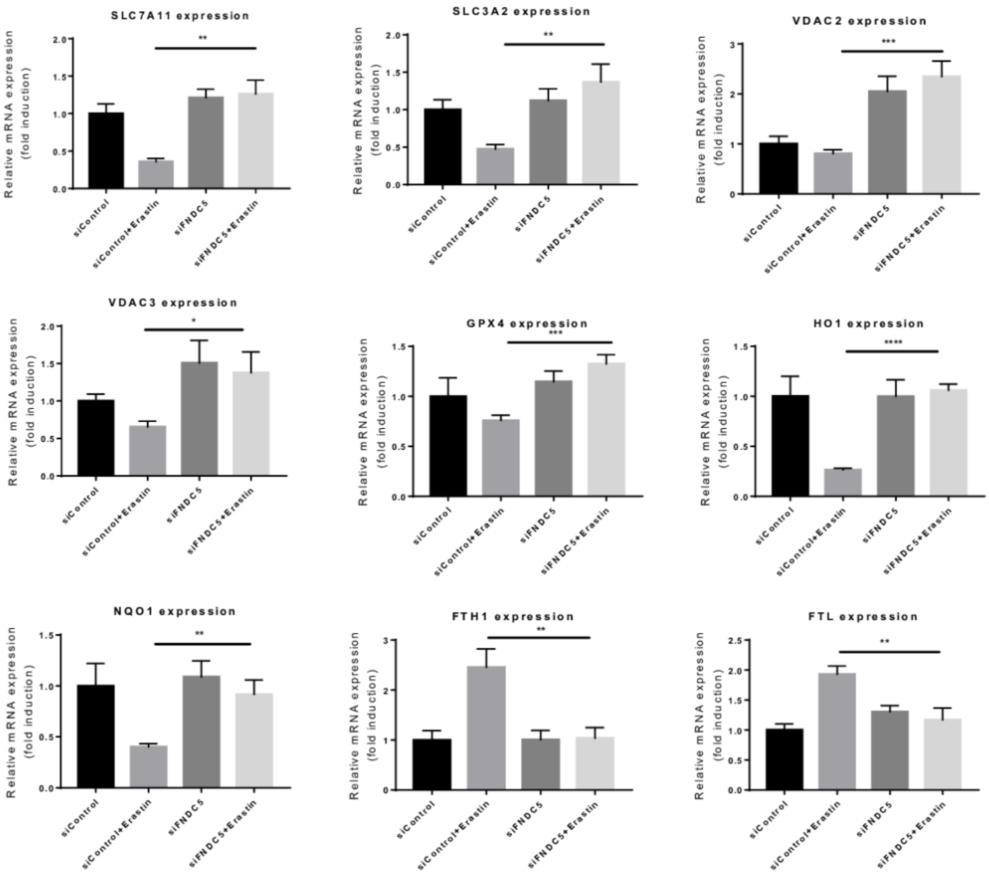

Figure S2. FNDC5 deletion was decreased the erastin-induced ferroptosis in PANC-1 cells. Ferroptosis related mRNA expression **(**system X_c^-, *SLC7A11, SLC3A2, GPX4*; ROS-*VDAC2, VDAC3, HO1, NQO1*; and iron metabolism, *FTL, FTH1***)** were changed after 12 h treatments with 20 μM erastin and/or 100 nM irisin. All data are expressed

as mean ± SEMs, $n \geqslant 3$; $^{**}p < .01$, $^{***}p < .001$$^{****}p < .0001$ vs. erastin group (paired t-tests).

Table S1. Polymerase chain reaction primer sequences.

Gene	Forward (5'→3')	Reverse (5'→3')
siFNDC5	CCCAAUAACAACAAGGAAATT	UUUCCUUGUUGUUAUUGGGTT
siControl	UUCUCCGAACGUGUCACGU	ACGUGACACGUUCGGAGAA
Human ACTB	TGTCCACCTTCCAGCAGATGT	CGGACTCGTCATACTCCTGCTT
Human SLC7A11	TGGGTGGAACTGCTCGTAAT	AGGATGTAGCGTCCAAATGC
Human SLC3A2	CTCGTGGTTCTCCACTCAGG	CCGCAATCAAGAGCCTGTCT
Human VDAC2	TTGGTTACGAGGGCTGGC	CGAAGTTACTCCTTGTCAGCTTTG
Human VDAC3	CAGACCCTTCGACCAGGAGT	TTCGCAACCCCTAGACTTCAG
Human HO1	CCAGGCAGAGAATGCTGAGTTC	AAGACTGGGCTCTCCTTGTTGC
Human NQO1	CCTGCCATTCTGAAAGGCTGGT	TGATGGAAAGCACTGCCT
Human FTH1	AAGATGGGTGCCCCTGAAG	CCAGGGTGTGCTTGTCAAAGA
Human FTL	CAGCCTGGTCAATTTGTACCT	GCCAATTCGCGGAAGAAGTG
Human GPX4	ACGTGGCCTCGCAATGCGGCAAAA CTGA	TCAGTTTTGCCGCATTGCGAGGCCA CGT

Table S2 Antibodies used in western blotting.

**Antibody**	**Dilution**	**Host species**	**Supplier**
LC3	1:1000	Rabbit	Abcam
NRF2	1:1000	Rabbit	Santa Cruz
p62	1:1000	Rabbit	Cell Signaling
FNDC5	1:1000	Rabbit	Cell Signaling
Atg7	1:1000	Rabbit	Cell Signaling
Atg5	1:1000	Rabbit	Cell Signaling
Beclin1	1:1000	Rabbit	Abcam
NCOA4	1:1000	Mouse	Abcam
p-p38	1:1000	Rabbit	Cell Signaling
p38	1:1000	Rabbit	Cell Signaling
pERK	1:1000	Rabbit	Cell Signaling
ERK	1:1000	Rabbit	Cell Signaling
pJNK	1:1000	Rabbit	Cell Signaling
JNK	1:1000	Rabbit	Cell Signaling
HRP-anti-rabbit IgG	1:1000	Donkey	Amersham
HRP-anti-mouse IgG	1:1000	Sheep	GE Healthcare
