# Supplementary material for: Irisin Is a Positive Regulator for Ferroptosis in Pancreatic Cancer
Source: Mol Ther Oncolytics. 2020 Aug 5;18:457–66. doi: 10.1016/j.omto.2020.08.002 (PMC7475648; doi:10.1016/j.omto.2020.08.002)
Supplement: Document S2. Article plus Supplemental Information [file mmc2.pdf]

Molecular Therapy
Oncolytics
Original Article

Irisin Is a Positive Regulator for Ferroptosis in Pancreatic Cancer

Bao Chen Yang[1] and Po Sing Leung[1]

[1]School of Biomedical Sciences, Faculty of Medicine, The Chinese University of Hong Kong, Hong Kong, China

Regulated cell death by way of ferroptosis involves iron-dependent accumulation of cellular reactive oxygen species (ROS). Ferroptosis is attracting attention as a potential therapeutic target for cancer treatments without drug resistance. The relationship between irisin, a myokine involved in autophagy and ROS metabolism, and ferroptosis is unclear. In this study, we used erastin-induced ferroptosis in PANC-1 cells to examine potential interactions of irisin with ferroptosis. Using western blots and reverse transcriptase polymerase chain reactions, we found that irisin can further exacerbate erastin-induced upregulation in free iron, lipid ROS levels, and glutathione depletion, relative to cells treated with erastin only. Conversely, removal of irisin limited erastin effects. Furthermore, irisin modulation of ferroptosis was associated with the expression changes in molecules important for ROS metabolism, iron metabolism, and the cysteine/glutamate antiporter system (system X_c^-). These study findings suggest that irisin can act as a master factor of ferroptosis, and that potential implications for harnessing irisin-mediated ferroptosis for cancer treatment are warranted.

INTRODUCTION

Ferroptosis, a form of regulated cell death identified in 2012, differs from general autophagy, apoptosis, and necrosis in that it involves an accumulation of cellular reactive oxygen species (ROS), an increase in free iron, and the disappearance of mitochondrial ridges.[1,2] Although the physiological function of ferroptosis is unclear, it has garnered interest for its potential therapeutic value in oncology. For example, ferroptosis inducers (i.e., erastin, buthionine sulfoximine, and sorafenib) have been shown to improve the anticancer effects of p62 knockdown, which prevents NRF2 (nuclear factor erythroid 2-related factor 2) accumulation, in hepatocellular carcinoma cells.[3] Anticancer effects of ferroptosis inducers on ovarian, pancreatic, and renal cancers have also been reported.[4,5] Ferroptosis has been suggested to be a form of autophagic cell death because cancer cells treated with ferroptosis inducers express autophagy factors, such as LC3 (microtubule-associated proteins 1A/1B light chain 3B) and ATGs (autophagy-related proteins).[6]

Irisin, a proteolytic cleavage product of fibronectin type III domain-containing protein 5 (FNDC5), is a recently characterized multi-functional myokine with hormonal actions that is highly expressed in muscle, heart, and adipose tissues and that is released from myocytes in response to exercise.[7] Irisin has been reported to be involved in glucose and lipid homeostasis regulation, immune regulation, and several systemic diseases. Recently, irisin has been reported to be a key positive regulator of autophagy. Specifically, it can increase autophagy and autophagic flux by activating the AMPK (5′ AMP-activated protein kinase) signaling pathway, which has a protective effect against cardiac hypertrophy.[8] In addition, irisin can reduce hepatic lipid accumulation by restoring AMPK/mTOR-mediated autophagy.[9,10] With respect to cell survival and apoptosis, irisin has been reported to reduce lung cancer cell invasiveness via the phosphoinositide 3-kinase/Akt/Snail pathway and to induce apoptosis and increase breast cancer cell sensitivity to an anti-neoplastic drug.[11,12] With respect to involvement in metabolic diseases, irisin has been reported to alleviate oxygen-glucose deprivation-induced oxidative stress and inflammation via inhibition of the ROS/NLRP3 inflammatory signaling pathway.[13]

Given that both ferroptosis and irisin can affect cancer cell survival via mechanisms involving autophagy, we hypothesized that irisin may interact with ferroptosis. To test this hypothesis, we examined the effects on irisin-mediated autophagy-related factors and molecules in PANC-1 cells (a human pancreatic cancer cell line) subjected to erastin-induced ferroptosis. We thus examined irisin-erastin interactive effects on levels of Fe^{2+}, glutathione (GSH), and the lipid ROS product malondialdehyde (MDA). Because both irisin and ferroptosis can affect ROS levels and autophagy, we investigated irisin treatment effects on the expression levels of the ROS-related protein NRF2 and the autophagy-related protein LC3 during ferroptosis. We also examined irisin effects on transcription of the system cystine/glutamate antiporter (system X_c^-)-related genes *SLC7A11*, *SLC3A2*, and *GPX4.* Given that PANC-1 cells produce endogenous irisin, we further examined the effects of irisin removal with a small interfering RNA (siRNA) targeting the transcript of its precursor protein FNDC5. Finally, we used transmission electron microscopy (TEM) to examine the effects of irisin on morphological manifestations of ferroptosis. We predicted that we would find evidence supporting the hypothesis and that irisin can act as a positive regulator of

Received 18 April 2020; accepted 2 August 2020;
https://doi.org/10.1016/j.omto.2020.08.002.

Correspondence: Po Sing Leung, PhD, School of Biomedical Sciences, Faculty of Medicine, The Chinese University of Hong Kong, Shatin, N.T., Room 609A, 6/F, Lo Kwee-Seong Integrated Biomedical Sciences Building, Hong Kong, China.
E-mail: psleung@cuhk.edu.hk

This is an open access article under the CC BY license (http://creativecommons.org/licenses/by/4.0/).

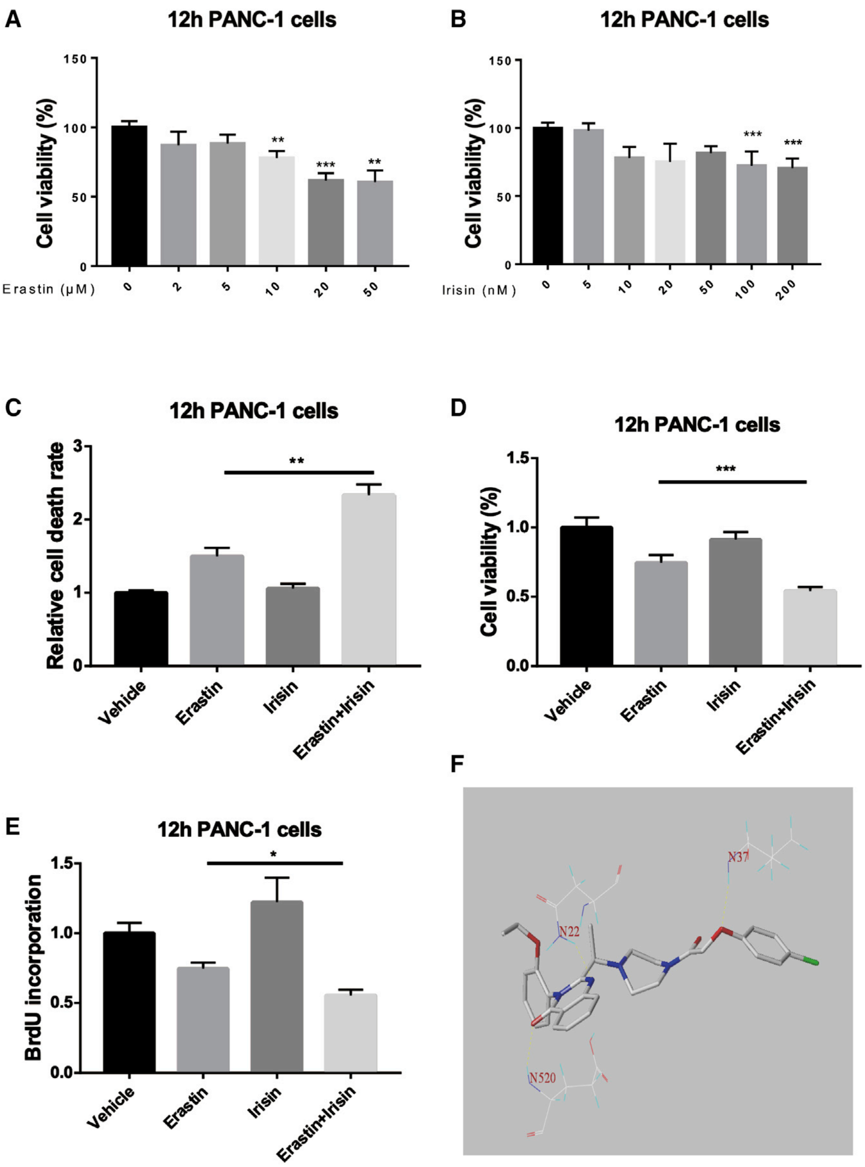

Figure 1. Irisin Increased Erastin-Induced Cell Death Rate and Decreased Cell Viability and Cell Proliferation

(A) Interaction of irisin (100 nM) and erastin (20 μM) effects on cell viability, cell death, and cell proliferation in PANC-1 cells compared to controls; 12 h of erastin treatment had a dose-dependent reducing effect on cell viability (n = 6/group). (B) At the same time, 12 h of 0–200 nM irisin treatment had a similar effect on 20 μM erastin-treated PANC-1 cells (n = 6/group). (C–E) Irisin increased cell death (C), decreased cell viability (D), and decreased cell proliferation (E) in PANC-1 cells subjected to erastin-induced ferroptosis. (F) With respect to docking of erastin to FNDC5, erastin showed a strong affinity for active site residues. All data are expressed as means ± SEM; n = 5/group. *p < 0.05, **p < 0.01, ***p < 0.001 versus the erastin group (paired t tests).

erastin-induced ferroptosis in cancer cells, likely via enhancement of ROS-mediated processes and autophagy.

RESULTS

Irisin Increases Erastin-Induced Ferroptosis in Pancreatic Cancer Cells

We observed a dose-dependent erastin-mediated induction of ferroptosis in PANC-1 cells assessed after 12 h of erastin exposure (Figure 1A). Co-treatment of PANC-1 cells with irisin and the ferroptosis inducer, erastin, together resulted in a greater cell death rate (Figure 1B), reduced cell viability (Figure 1C), and reduced cell proliferation (Figure 1D) relative to erastin-induced ferroptosis alone. Computational analysis yielded a docking interaction score for erastin and the irisin precursor FNDC5 of 7.5475; these data also indicated that erastin forms hydrogen bonds with a major catalytic site in FNDC5 at the key asparagine residues (N22, N37, and N520; Figure 1E).

Irisin Increases Ferroptosis-Associated Lipid ROS Accumulation, GSH Depletion, and Iron Accumulation

Co-treatment with irisin and erastin for 12 h resulted in a significant upregulation of the levels for lipid ROS levels (Figure 2A) and total ROS (Figure 2B), as well as significantly increased levels of the ROS product MDA (Figure 2C) and Fe^{2+} (Figure 2D), compared to erastin alone. Co-treatment with irisin and erastin for 12 h also resulted in significantly reduced levels of GSH (Figure 2E), compared to erastin alone. We also observed a dose-dependent effect of irisin on GSH levels in PANC-1 cells in the concentration range of 0–100 nM (Figure 2F).

Irisin Decreases the Expression of NRF2 and LC3 during Ferroptosis

We observed dose- and time-dependent effects of irisin on the expression of NRF2 and LC3 (Figures 3A and 3B). Meanwhile, there was a dramatic downregulation of p62 expression, which inhibited NRF2 degradation and enhanced NRF2 nuclear accumulation after 12 h of irisin and erastin co-treatment, although irisin or erastin alone did not affect p62 levels (Figure 3C). Levels of the autophagy-related proteins, i.e., Atg5, Atg7 and Beclin, in the irisin and erastin co-treated group were similar to the respective levels of these proteins in the erastin-treated group (Figures S1A and S1B). Transcript levels of the system X_c^--related genes *SLC7A11*, *SLC3A2*, and *GPX4* in the irisin and erastin co-treated group were lower whereas transcript levels of the iron metabolism-related genes *FTH1* and *FTL* were higher than respective transcript levels in the erastin-treated group. Although the co-treatment did not alter mitogen-activated protein kinase (MAPK) expression, relative to erastin-

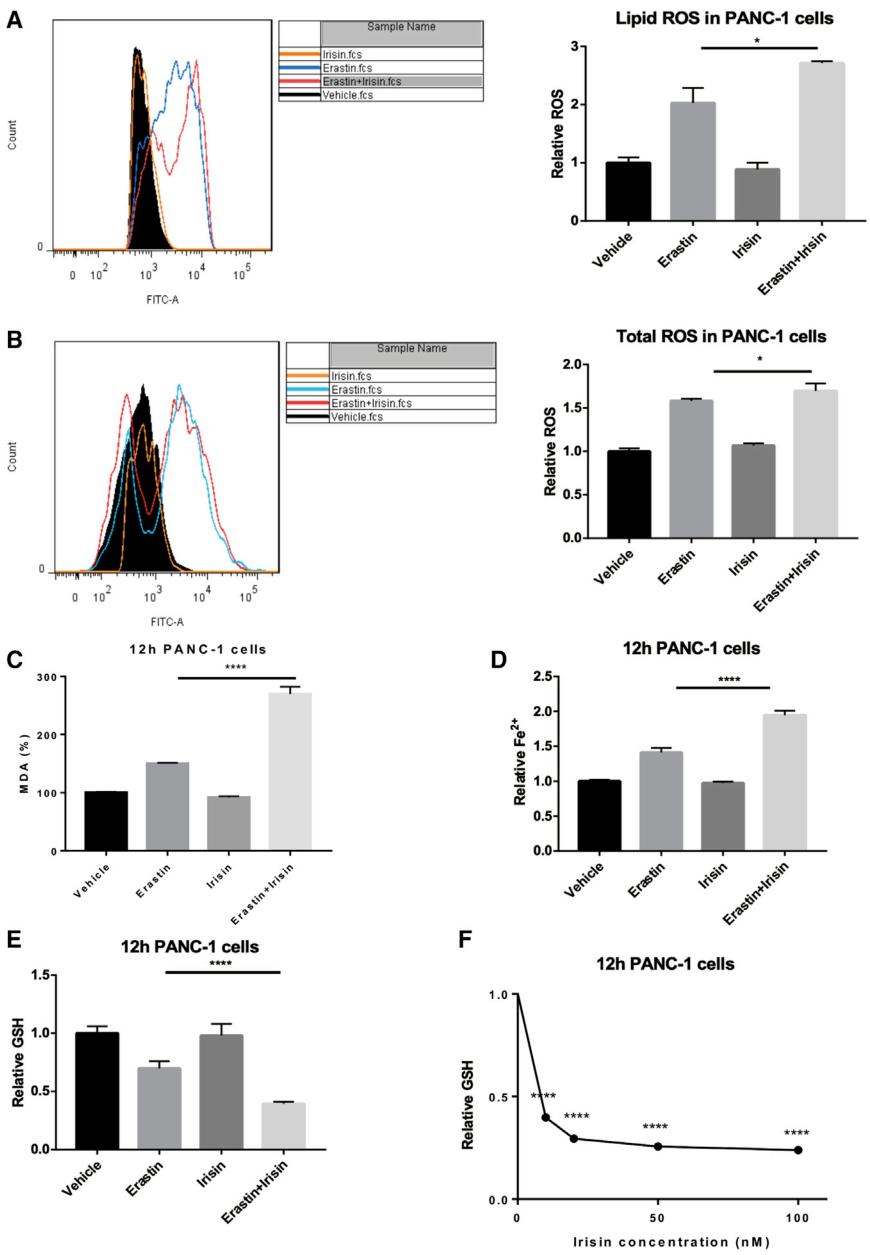

Figure 2. Irisin Effects on Lipid and Iron Metabolism

(A–E) Irisin treatment (100 nM, 12 h) resulted in significant enhancements of 20 μM erastin effects on ferroptosis-associated lipid ROS levels (A), total ROS levels (B), MDA percentage (C), Fe^{2+} concentrations (D), and relative GSH concentrations (E). All data in (A)–(E) are expressed as means ± SEM; n = 3–5/group. *$p < 0.05$, ****$p < 0.0001$ versus the erastin group (paired t tests). (F) Relative GSH concentration in response to a range of irisin concentrations in the presence of 20 μM erastin for 12 h; n = 5/concentration.

treated cells, it did increase mRNA levels of the ROS metabolism-related genes encoding voltage-dependent anion channel (*VDAC*)*2*, *VDAC3*, heme oxygenase 1 (*HO1*), and NADPH (reduced nicotinamide adenine dinucleotide phosphate) quinone dehydrogenase 1 (*NQO1*) (Figure 1C).

Suppression of Irisin Downregulates Erastin-Induced Cell Death

After suppression of irisin expression in PANC-1 cells with a siRNA targeting its precursor transcript, we observed marked reduction in cell death rate and apoptosis (Figures 4A and AB), with a concomitant increase in cell proliferation (Figure 4C). Moreover, TEM examination demonstrated that the typical features of ferroptosis were attenuated following siRNA-mediated suppression of irisin expression, which included the ferroptosis-specific features of disappearance of mitochondrial cristae, plasma membrane blebbing and a lack of chromatin condensation in nuclei, as well as an increased presence of autophagosomes (Figure 4D).

Suppression of Irisin Blocks Erastin-Induced Ferroptosis-Associated ROS and GSH Depletion and Iron Accumulation

In PANC-1 cells subjected to erastin-induced ferroptosis, siRNA suppression of endogenous irisin expression resulted in increased cell viability and relative GSH concentrations (Figures 5A and 5B) together with reduced levels of accumulated Fe^{2+}, lipid ROS, and the lipid ROS product MDA, relative to ferroptotic control cells (Figures 5C–5E). Furthermore, cells in which irisin was suppressed exhibited normal levels of NRF2 and LC3 (Figure 5F) as well as a normalization of system X_c^--related factors (*SLC7A11*, *SLC3A2*, and *GPX4*), ROS metabolism-related factors (*VDAC2*, *VDAC3*, *HO1*, and *NQO1*), and iron metabolism-related factors (*FTH1* and *FTL*) (Figure 6; Figure S2).

DISCUSSION

Pancreatic cancer was the fourth leading cause of cancer-related death in developed countries in 2010. It is projected to exceed the numbers of breast, prostate, and colorectal cancers and become the second leading cause of cancer-related morbidity and mortality by 2030.[14] Pancreatic cancer patients easily produce resistance to chemotherapy and radiotherapy, which explains the high death rate. Given that several ferroptosis-inducing agents are already US Food and Drug Administration (FDA)-approved drugs, including erastin, ferroptosis represents a promising therapeutic strategy for curing difficult-to- treat cancers,[14] including pancreatic cancer, which is highly resistant to chemotherapy drugs and has the worst survival rate of all cancers.[15,16] The present results extend previous research

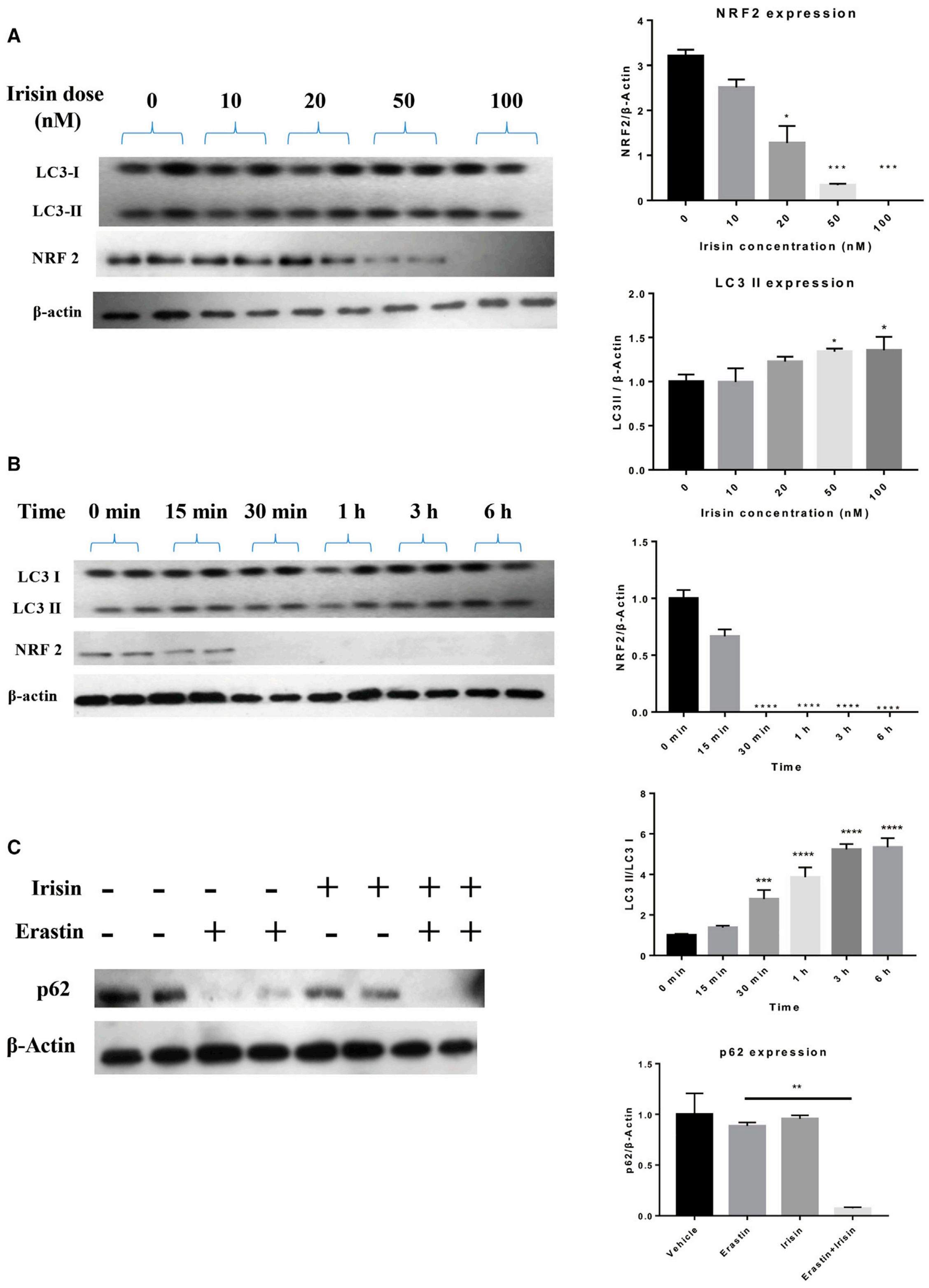

(legend on next page)

showing that irisin can inhibit the proliferation, migration, and invasion of cancer cells while increasing cancer cell death by regulating ROS metabolism and autophagy.[17,18] Consistent with our hypothesis, the present experiments showed that irisin interacts with numerous effects of a ferroptosis inducer on cancer cells. We observed that irisin can augment ferroptosis-associated increases in free iron concentrations, lipid peroxide levels, and GSH depletion, while increasing the pancreatic cancer cell death rate.

NRF2 has been described as a master regulator of lipid peroxidation metabolism, and NRF2 dysfunction is an important feature of ferroptosis.[19] Increasing NRF2 expression can promote cell survival, while decreasing NRF2 expression can increase cell death. Interestingly, we found in the present study that irisin-erastin co-treatment significantly increased NRF2 expression and lipid ROS levels relative to erastin-treated cells not exposed to irisin. Previously, Sun at al.[3] reported downregulation of NRF2 expression in hepatocellular carcinoma cells exposed to erastin, and argued that this NRF2 downregulation reflects the non-sensitivity of hepatocellular carcinoma cells to ferroptosis. If so, the presently observed opposite result with PANC-1 cells would suggest that human pancreatic cancer cells should be sensitive to ferroptosis. Consistent with this deduction, we showed in this study that p62, which prevents NRF2 degradation,[20] almost disappeared in cells exposed to irisin-erastin co-treatment, while remaining high in erastin-treated cells not exposed to irisin. Moreover, suppression of irisin expression blocked numerous erastin-induced effects in PANC-1 cells, suggesting that irisin may be an essential factor in ferroptotic lipid peroxidation rather than simply an augmenter of cell sensitivity to ferroptotic induction.

There is an essential link between NRF2 and iron metabolism. NRF2 affects the expression of two iron storage proteins, FTL and FTH1,[21,22] and has also been shown to affect the SLC7A11 and SLC3A2 chains of the cysteine/glutamate transporter xCT, which is important for GSH synthesis.[23,24] Ferroptosis induction is associated with upregulation of cellular free Fe^{2+} levels and increased transcription of *FTH1*.[6] In this study, we showed that irisin increased free Fe^{2+} levels and *FTL* and *FTH1* transcript levels in ferroptotic PANC-1 cells significantly. Furthermore, we found that GSH levels and the expression of the SLC7A11 and SLC3A2 chains of the cysteine/glutamate transporter xCT were significantly downregulated in irisin-treated ferroptotic PANC-1 cells, compared to expression in ferroptotic PANC-1 cells not exposed to erastin. Quite remarkably, suppression of irisin expression blocked these changes. It has been known that GPX4 is an essential regulator of ferroptosis and its inhibition increases lipid peroxidation,[25] which is regulated by NRF2.[26] Therefore, we probed the effects of irisin on GPX4 expression in the context of ferroptosis, and obtained convergent results regarding irisin effects on GPX4 in erastin-induced ferroptosis.

A particular form of autophagy, termed ferritinophagy, mediates a crucial role in regulating ferroptosis via induction of cellular iron stock protein degradation to increase free iron concentrations via the NCOA4 autophagy pathway.[27–29] In this study, we observed that erastin-induced ferroptosis-associated reduction in NCOA4 expression was blocked in the absence of irisin. Moreover, our TEM experimental data showed that irisin suppression resulted in substantial reductions in autophagosome numbers together with a blockade of the hallmark cytomorphological features of ferroptosis.

In conclusion, the present findings demonstrate that irisin is a key factor in ferroptosis. Collectively, our results support pursuing pharmacological ferroptosis inducers (several of which already have FDA approval, including erastin) as potentially curative treatments for difficult-to-treat cancers, and pancreatic cancer in particular, with irisin as a potential adjunct treatment enhancer. Endogenous irisin, which is upregulated in response to short bouts of intensive exercise, has been related to metabolic and immune regulation, and has anti-inflammatory effects.[11,30] However, serum irisin concentrations of cancer patients, who often are not exercising, are consistently lower than concentrations in healthy people.[31] We thus advocate that the potential for using exercise-induced upregulation of irisin and/or exogenous irisin administration in combination with ferroptosis-induction therapy (e.g., with erastin) should be explored intensively.

MATERIALS AND METHODS

Cell Culture and Transfection

The human pancreatic cancer cell line PANC-1 was purchased from the American Type Culture Collection (Manassas, VA, USA). The cells were cultured in T75-cm^2 plastic flasks and maintained in Dulbecco's modified Eagle's medium (DMEM) containing 10% (v/v) fetal bovine serum (FBS) with 100 U/mL penicillin, and incubated at 37°C in a 5% CO_2, 95% humidified atmosphere. Anti-human *FNDC5* siRNA and scrambled siRNA (sequences in Table S1) were synthesized and transfected into PANC-1 cells with Lipofectamine RNAi Max (Invitrogen, catalog no. 13778150) for 48 h according to the manufacturer's instructions. FNDC5-suppressed cells were treated with or without erastin (Sigma) as indicated.

Polymerase Chain Reaction Analyses

Total RNA was extracted from PANC-1 cells with TRIzol reagent (Invitrogen) according to the manufacturer's instructions. Reverse transcriptase (RT) of first-strand cDNA was performed with a PrimeScript RT master mix kit (Takara Bio, Kusatsu, Japan). Gene

Figure 3. Irisin Increase Erastin-Induced Autophagic Protein Expression and NRF2 Expression

Dose and time dependency of irisin effects on erastin-induced ferroptosis, as evidenced by changes in LC3II and NRF2 expression, and the irisin plus erastin effect on p65 in PANC-1 cells are shown. (A) Dose-dependent effects of irisin in the presence of 20 μM erastin (12-h treatment). (B) Time-dependent effects of irisin (100 nM) in the presence of 20 μM erastin. Data in (A) and (B) are means ± SEM; $n \geq 3$. *$p < 0.05$, ***$p < 0.001$, ****$p < 0.0001$ versus the vehicle group (one-way ANOVA). (C) Western blot analysis with anti-p62 and anti-β-actin antibodies of PANC-1 cells lysed after 12-h treatments (20 μM erastin and/or 100 nM irisin, 12 h). The data are means ± SEM; $n \geq 3$. **$p < 0.01$ versus the erastin group (paired t tests).

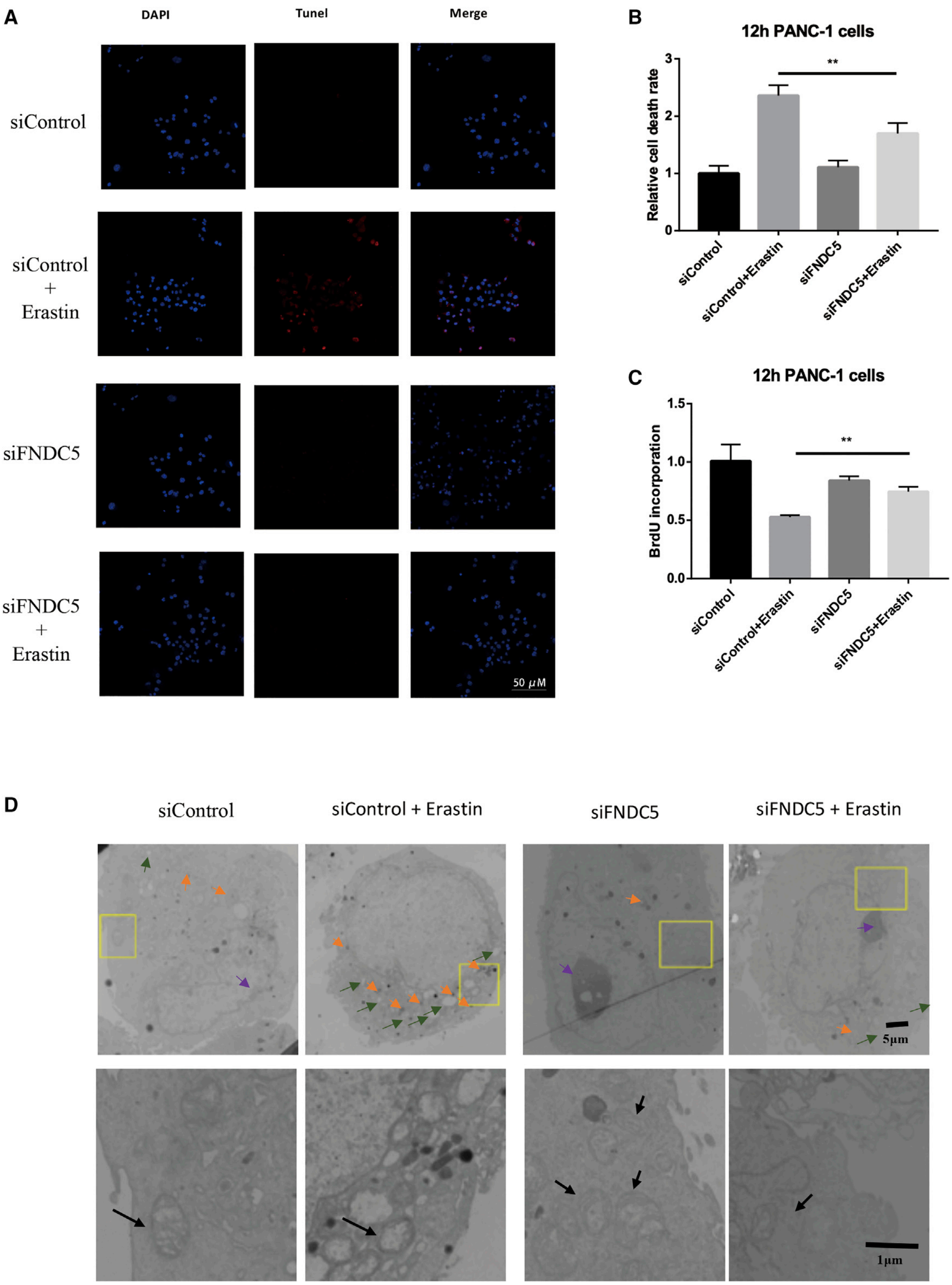

Figure 4. Ferroptotic Inducers Have No Effect on PANC-1 Cells after Knockdown of FNDC5 in Morphology

Suppression of the expression of irisin with a siRNA targeting the transcript of its precursor FNDC5 decreased the effects of 20 μM, 12-h erastin-induced ferroptosis on cell death rate, cell proliferation, and cytomorphology. (A) Representative images by group of immunofluorescence-labeled apoptotic PANC-1 cells. Scale bar, 50 μm. (B and C) Decreased cell death rate (B) and increased cell proliferation (C) in the siRNA-treated group. All data are expressed as means ± SEM; n = 5/group. $^{*}p < 0.05$, $^{**}p < 0.01$, $^{****}p <$

(legend continued on next page)

expression was measured by conventional or real-time polymerase chain reaction (PCR), wherein cDNA samples were mixed with SYBR Green qPCR master mix (Applied Biosystems, Waltham, MA, USA) and specific primers (Table S1). The fold change in mRNA expression relative to the control group was determined with the $2^{-\Delta\Delta Ct}$ method and normalized to β-actin mRNA levels.

Western Blot Analysis

PANC-1 cell proteins were extracted with radioimmunoprecipitation assay (RIPA) buffer. Extracted proteins were separated by 8%–12% sodium dodecyl sulfate-polyacrylamide gel electrophoresis and transferred to nitrocellulose membranes (Bio-Rad, Heidemannstrasse, Germany), which were blocked with 5% milk and then incubated overnight with primary antibodies at 4°C. Horseradish peroxidase (HRP)-conjugated secondary antibodies were incubated at room temperature with membranes for 2 h after washing with phosphate-buffered saline (PBS) with 0.1% Tween 20. Labeled protein bands were visualized on autoradiography films (Fuji Film, Tokyo, Japan) following application of enhanced chemiluminescence (ECL) detection reagent (GE Healthcare, Chicago, IL, USA). Protein bands were quantitated in ImageJ software (National Institutes of Health, Bethesda, MD, USA) and normalized to β-actin. The primary and secondary antibodies used are listed in Table S2.

Cell Viability Assay

Cellular metabolic activity was measured with a 3-(4,5-dimethyl-2-thiazolyl)-2,5-diphenyltetrazolium (MTT) bromide assay immediately after a 24-h ethanol treatment. The cells were seeded in a 96-well cell culture plates at 4×10^4 cells/well for 1 day and then incubated in 2% FBS media with or without ethanol for 24 h. Subsequently, 0.15 mg of MTT was added to each well. After the medium was discarded, the cells were allowed to incubate for 3 h at 37°C, and then 100 μL of dimethyl sulfoxide was added to each well and the cells were left for 15 min at room temperature. Finally, optical density at 490 nm was read in a microplate reader (SpectraMax i3x multi-mode detection platform, Molecular Devices, CA, USA). Cell viability was expressed as a percentage of the quantity of cells observed in the non-ethanol medium control group.

Cell Death Rate and Cell Proliferation Rate

Cells were seeded at a density of 4×10^4 cells/well in a 96-well cell culture plate and cultured under experimentally indicated conditions. Cell death rate was determined by a cell death detection ELISA plus kit (Roche Applied Science) according to the manufacturer's instructions. Cell proliferation was determined by detecting bromodeoxyuridine (BrdU) incorporation. After cells were incubated with BrdU for 2 h, DNA synthesis was assessed with a BrdU cell proliferation ELISA kit (Roche Applied Science, Basel, Switzerland).

TUNEL

TUNEL (terminal deoxynucleotidyltransferase-mediated deoxyuridine triphosphate nick end labeling) assays were performed with a one-step TUNEL apoptosis assay kit (Beyotime, China) according to the manufacturer's instructions.

Total ROS and Lipid ROS Measurement

Cells were incubated for 1 h with 10 μM 2′,7′-dichlorodihydrofluorescein diacetate (H2DCFDA, Life Technologies) for total ROS determination or 10 μM C11-BODIPY (Thermo Fisher Scientific) for lipid ROS determination, and then rinsed twice in PBS. Labeled cells were harvested with trypsin and suspended in PBS with 5% FBS. ROS levels were analyzed with a flow cytometer (LSRFortessa, BD Biosciences) in a minimum of 10,000 cells per condition.

Iron, GSH, and Lipid Peroxidation Assays

Relative iron, cell-lysate GSH, and relative MDA concentrations were analyzed with an iron colorimetric assay kit (BioVision, Milpitas, CA, USA), a GSA assay kit (Beyotime, China), and lipid peroxidation assay kit (Beyotime, China), respectively, according to the manufacturers' protocols.

TEM

Cells were digested in 0.25% trypsin, fixed with 2.5% glutaraldehyde phosphate (0.1 M, pH 7.4) for 30 min, postfixed in 2% aqueous osmium tetroxide, dehydrated in graded ethanol solutions (70%–100%) with propylene oxide, embedded in Epon 812 (Merck), and cured for 48 h at 60°C. Semithin sections (1.0 μm) were cut and stained with toluidine blue. Ultrathin sections (80 nm) were collected onto 200-mesh copper grids and stained with uranyl acetate and lead citrate before examination by TEM with an H-7700 microscope (Hitachi, Tokyo, Japan).

Molecular Modeling

Computational studies, including erastin-FNDC5 docking analysis, were performed in SYBYL-X 2.1.1; the crystal structure of human FNDC5 (PDB: 4LSD) was retrieved from the Protein Data Bank (https://www.rcsb.org/). Structures were determined with Tripos force field and MMFF99 charges according to the Powell method (termination: energy change of 0.01 kcal/mol A° with a maximum of 100,000 iterations).

Statistical Analysis

All data were obtained from at least three independent experiments. Data are expressed as means ± standard errors of the mean (SEM). Statistical significance was determined with Student's t tests and analyses of variance (ANOVAs).

SUPPLEMENTAL INFORMATION

Supplemental Information can be found online at https://doi.org/10.1016/j.omto.2020.08.002.

0.0001 versus siFNDC5 + erastin group (paired t tests). (D) TEM of FNDC5-devoid PANC-1 cells treated with erastin (12 h). Orange arrowheads indicate autophagosomes; black arrowheads indicate mitochondria; green arrowheads indicate blebs; purple arrowheads indicate chromatin. The images in the bottom row (scale bar, 1 μm) are magnified views of areas enclosed by yellow rectangles in the images immediately above (scale bar, 5 μm).

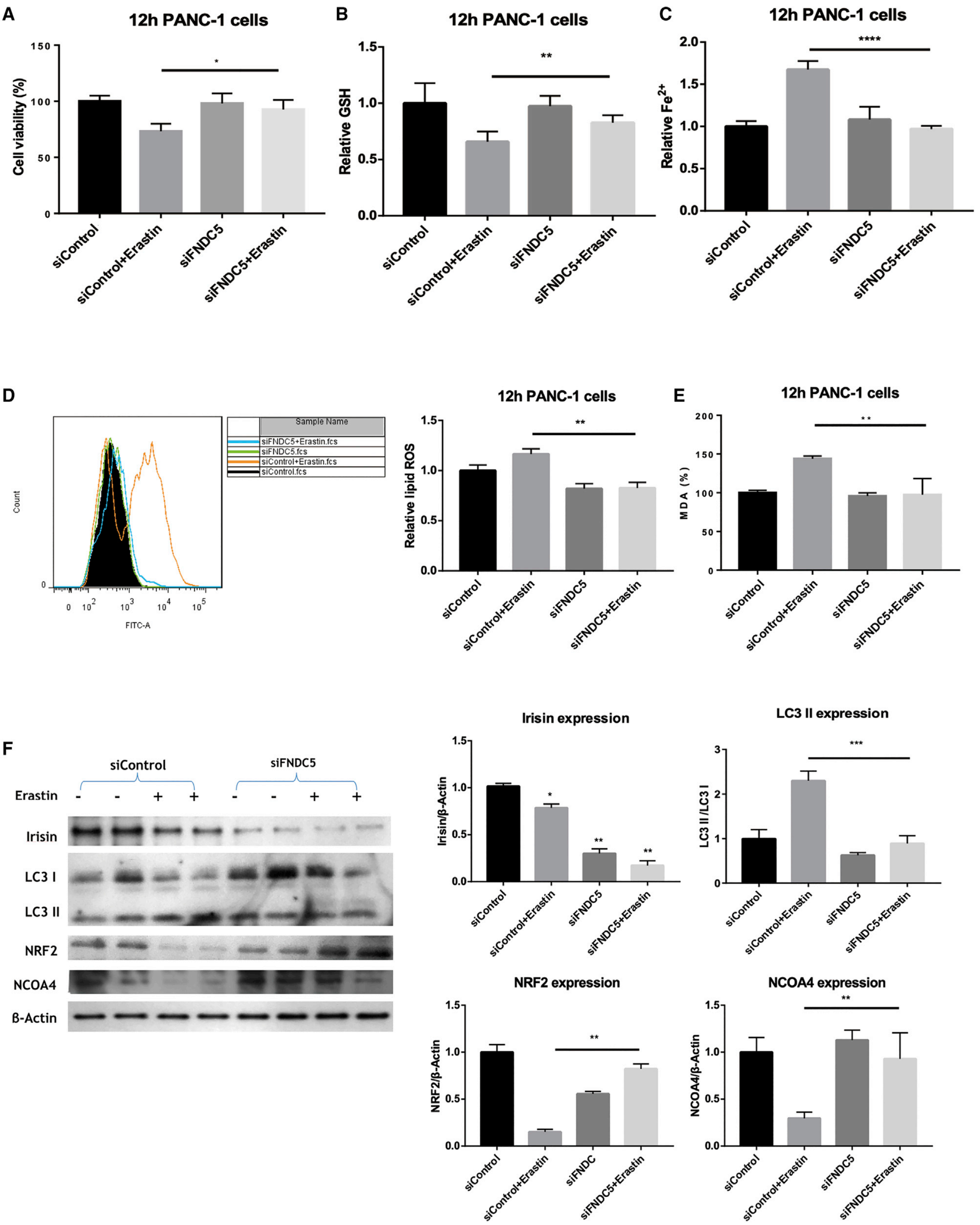

(legend on next page)

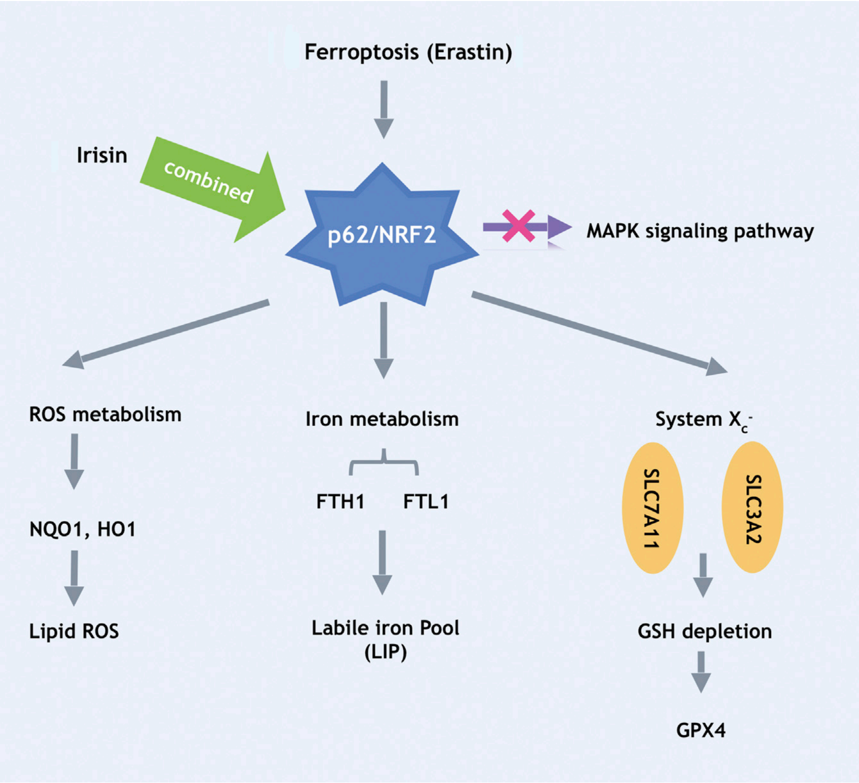

Figure 6. Schematic Diagram Summarizing the Regulatory Actions of Irisin and Erastin in ROS Metabolism, Iron Metabolism, and System X_c^- Molecules

AUTHOR CONTRIBUTIONS

Y.B.C.: design, collection, and/or assembly of data, data analysis, and manuscript writing. P.S.L: conception and design, provision of study material, manuscript writing, final approval of manuscript, and financial support.

CONFLICTS OF INTEREST

The authors declare no competing interests.

ACKNOWLEDGMENTS

This work was supported by the Chinese University of Hong Kong (no. CUHK7104280, awarded to P.S.L.).

REFERENCES

1. Dixon, S.J., Lemberg, K.M., Lamprecht, M.R., Skouta, R., Zaitsev, E.M., Gleason, C.E., Patel, D.N., Bauer, A.J., Cantley, A.M., Yang, W.S., et al. (2012). Ferroptosis: an iron-dependent form of nonapoptotic cell death. Cell *149*, 1060–1072.

2. Xie, Y., Hou, W., Song, X., Yu, Y., Huang, J., Sun, X., Kang, R., and Tang, D. (2016). Ferroptosis: process and function. Cell Death Differ. *23*, 369–379.

3. Sun, X., Ou, Z., Chen, R., Niu, X., Chen, D., Kang, R., and Tang, D. (2016). Activation of the p62-Keap1-NRF2 pathway protects against ferroptosis in hepatocellular carcinoma cells. Hepatology *63*, 173–184.

4. Eling, N., Reuter, L., Hazin, J., Hamacher-Brady, A., and Brady, N.R. (2015). Identification of artesunate as a specific activator of ferroptosis in pancreatic cancer cells. Oncoscience *2*, 517–532.

5. Kim, S.E., Zhang, L., Ma, K., Riegman, M., Chen, F., Ingold, I., Conrad, M., Turker, M.Z., Gao, M., Jiang, X., et al. (2016). Ultrasmall nanoparticles induce ferroptosis in nutrient-deprived cancer cells and suppress tumour growth. Nat. Nanotechnol. *11*, 977–985.

6. Gao, M., Monian, P., Pan, Q., Zhang, W., Xiang, J., and Jiang, X. (2016). Ferroptosis is an autophagic cell death process. Cell Res. *26*, 1021–1032.

7. Natalicchio, A., Marrano, N., Biondi, G., Spagnuolo, R., Labarbuta, R., Porreca, I., Cignarelli, A., Bugliani, M., Marchetti, P., Perrini, S., et al. (2017). The myokine irisin is released in response to saturated fatty acids and promotes pancreatic β-cell survival and insulin secretion. Diabetes *66*, 2849–2856.

Figure 5. Knockdown of FNDC5 Avoids the Effects of Erastin on Lipid and Iron Metabolism

FNDC5-targeting siRNA alters ferroptosis-related parameters and the expression of autophagy and ferroptosis-related proteins in PANC-1 cells. (A–E) Relative cell viability (A) and GSH depletion (B) of in the irisin-suppressed (with FNDC5 siRNA) group were increased compared to the siControl + erastin group, as were Fe^{2+} concentration (C), lipid ROS levels (D), and MDA concentrations (E). The data are means ± SEM; n = 5/group. *p < 0.05, **p < 0.01, ****p < 0.0001. (F) Expression levels of autophagy-related protein LC3II and the ferroptosis-related protein NRF2 were downregulated and upregulated, respectively, in the siFNDC5 + erastin group compared to the siControl + erastin group. Ferroptosis was induced with 20 μM erastin for 12 h. The data are means ± SEM; n = 3/group. *p < 0.05, **p < 0.01, ***p < 0.001 versus the siFNDC5 + erastin group (paired t tests).

8. Zhang, D., Xie, T., and Leung, P.S. (2018). Irisin ameliorates glucolipotoxicity-associated β-cell dysfunction and apoptosis via AMPK signaling and anti-inflammatory actions. Cell. Physiol. Biochem. *51*, 924–937.

9. Li, R.L., Wu, S.S., Wu, Y., Wang, X.X., Chen, H.Y., Xin, J.J., Li, H., Lan, J., Xue, K.Y., Li, X., et al. (2018). Irisin alleviates pressure overload-induced cardiac hypertrophy by inducing protective autophagy via mTOR-independent activation of the AMPK-ULK1 pathway. J. Mol. Cell. Cardiol. *121*, 242–255.

10. Liu, T.Y., Xiong, X.Q., Ren, X.S., Zhao, M.X., Shi, C.X., Wang, J.J., Zhou, Y.B., Zhang, F., Han, Y., Gao, X.Y., et al. (2016). FNDC5 alleviates hepatosteatosis by restoring AMPK/mTOR-mediated autophagy, fatty acid oxidation, and lipogenesis in mice. Diabetes *65*, 3262–3275.

11. Shao, L., Li, H., Chen, J., Song, H., Zhang, Y., Wu, F., Wang, W., Zhang, W., Wang, F., Li, H., and Tang, D. (2017). Irisin suppresses the migration, proliferation, and invasion of lung cancer cells via inhibition of epithelial-to-mesenchymal transition. Biochem. Biophys. Res. Commun. *485*, 598–605.

12. Gannon, N.P., Vaughan, R.A., Garcia-Smith, R., Bisoffi, M., and Trujillo, K.A. (2015). Effects of the exercise-inducible myokine irisin on malignant and non-malignant breast epithelial cell behavior in vitro. Int. J. Cancer *136*, E197–E202.

13. Peng, J., Deng, X., Huang, W., Yu, J.H., Wang, J.X., Wang, J.P., Yang, S.B., Liu, X., Wang, L., Zhang, Y., et al. (2017). Irisin protects against neuronal injury induced by oxygen-glucose deprivation in part depends on the inhibition of ROS-NLRP3 inflammatory signaling pathway. Mol. Immunol. *91*, 185–194.

14. Zhai, B., and Sun, X.Y. (2013). Mechanisms of resistance to sorafenib and the corresponding strategies in hepatocellular carcinoma. World J. Hepatol. *5*, 345–352.

15. Long, J., Zhang, Y., Yu, X., Yang, J., LeBrun, D.G., Chen, C., Yao, Q., and Li, M. (2011). Overcoming drug resistance in pancreatic cancer. Expert Opin. Ther. Targets *15*, 817–828.

16. Ansari, D., Tingstedt, B., Andersson, B., Holmquist, F., Sturesson, C., Williamsson, C., Sasor, A., Borg, D., Bauden, M., and Andersson, R. (2016). Pancreatic cancer: yesterday, today and tomorrow. Future Oncol. *12*, 1929–1946.

17. Moon, H.S., and Mantzoros, C.S. (2014). Regulation of cell proliferation and malignant potential by irisin in endometrial, colon, thyroid and esophageal cancer cell lines. Metabolism *63*, 188–193.

18. Askari, H., Rajani, S.F., Poorebrahim, M., Haghi-Aminjan, H., Raeis-Abdollahi, E., and Abdollahi, M. (2018). A glance at the therapeutic potential of irisin against diseases involving inflammation, oxidative stress, and apoptosis: an introductory review. Pharmacol. Res. *129*, 44–55.

19. Dodson, M., Castro-Portuguez, R., and Zhang, D.D. (2019). NRF2 plays a critical role in mitigating lipid peroxidation and ferroptosis. Redox Biol. *23*, 101107.

20. Bae, S.H., Sung, S.H., Oh, S.Y., Lim, J.M., Lee, S.K., Park, Y.N., Lee, H.E., Kang, D., and Rhee, S.G. (2013). Sestrins activate Nrf2 by promoting p62-dependent autophagic degradation of Keap1 and prevent oxidative liver damage. Cell Metab. *17*, 73–84.

21. Agyeman, A.S., Chaerkady, R., Shaw, P.G., Davidson, N.E., Visvanathan, K., Pandey, A., and Kensler, T.W. (2012). Transcriptomic and proteomic profiling of KEAP1 disrupted and sulforaphane-treated human breast epithelial cells reveals common expression profiles. Breast Cancer Res. Treat. *132*, 175–187.

22. Harada, N., Kanayama, M., Maruyama, A., Yoshida, A., Tazumi, K., Hosoya, T., Mimura, J., Toki, T., Maher, J.M., Yamamoto, M., and Itoh, K. (2011). Nrf2 regulates ferroportin 1-mediated iron efflux and counteracts lipopolysaccharide-induced ferroportin 1 mRNA suppression in macrophages. Arch. Biochem. Biophys. *508*, 101–109.

23. Ishii, T., Itoh, K., Takahashi, S., Sato, H., Yanagawa, T., Katoh, Y., Bannai, S., and Yamamoto, M. (2000). Transcription factor Nrf2 coordinately regulates a group of oxidative stress-inducible genes in macrophages. J. Biol. Chem. *275*, 16023–16029.

24. Chen, D., Tavana, O., Chu, B., Erber, L., Chen, Y., Baer, R., and Gu, W. (2017). NRF2 is a major target of ARF in p53-independent tumor suppression. Mol. Cell *68*, 224–232.e4.

25. Yang, W.S., SriRamaratnam, R., Welsch, M.E., Shimada, K., Skouta, R., Viswanathan, V.S., Cheah, J.H., Clemons, P.A., Shamji, A.F., Clish, C.B., et al. (2014). Regulation of ferroptotic cancer cell death by GPX4. Cell *156*, 317–331.

26. Singh, A., Rangasamy, T., Thimmulappa, R.K., Lee, H., Osburn, W.O., Brigelius-Flohé, R., Kensler, T.W., Yamamoto, M., and Biswal, S. (2006). Glutathione peroxidase 2, the major cigarette smoke-inducible isoform of GPX in lungs, is regulated by Nrf2. Am. J. Respir. Cell Mol. Biol. *35*, 639–650.

27. Dowdle, W.E., Nyfeler, B., Nagel, J., Elling, R.A., Liu, S., Triantafellow, E., Menon, S., Wang, Z., Honda, A., Pardee, G., et al. (2014). Selective VPS34 inhibitor blocks autophagy and uncovers a role for NCOA4 in ferritin degradation and iron homeostasis in vivo. Nat. Cell Biol. *16*, 1069–1079.

28. Mancias, J.D., Wang, X., Gygi, S.P., Harper, J.W., and Kimmelman, A.C. (2014). Quantitative proteomics identifies NCOA4 as the cargo receptor mediating ferritinophagy. Nature *509*, 105–109.

29. Hou, W., Xie, Y., Song, X., Sun, X., Lotze, M.T., Zeh, H.J., 3rd, Kang, R., and Tang, D. (2016). Autophagy promotes ferroptosis by degradation of ferritin. Autophagy *12*, 1425–1428.

30. Löffler, D., Müller, U., Scheuermann, K., Friebe, D., Gesing, J., Bielitz, J., Erbs, S., Landgraf, K., Wagner, I.V., Kiess, W., and Körner, A. (2015). Serum irisin levels are regulated by acute strenuous exercise. J. Clin. Endocrinol. Metab. *100*, 1289–1299.

31. Provatopoulou, X., Georgiou, G.P., Kalogera, E., Kalles, V., Matiatou, M.A., Papapanagiotou, I., Sagkriotis, A., Zografos, G.C., and Gounaris, A. (2015). Serum irisin levels are lower in patients with breast cancer: association with disease diagnosis and tumor characteristics. BMC Cancer *15*, 898.

OMTO, Volume *18*

Supplemental Information

Irisin Is a Positive Regulator for Ferroptosis in Pancreatic Cancer

Bao Chen Yang and Po Sing Leung

Irisin is a positive regulator for ferroptosis in pancreatic cancer

Bao Chen Yang and Po Sing Leung*

Supplementary data

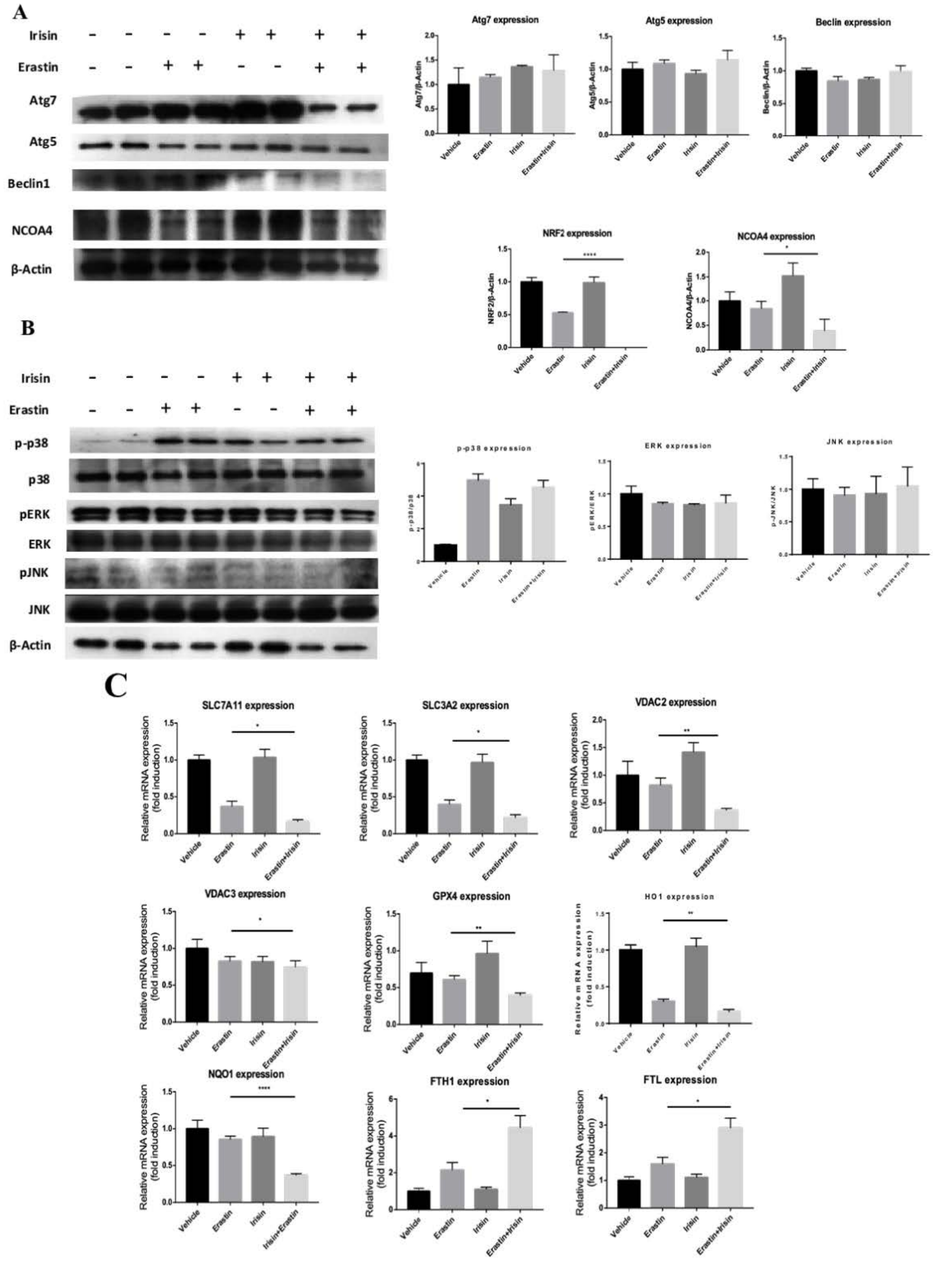

Figure S1. Irisin effects on autophagy-related proteins in ferroptotic PANC-1 cells. (A) Protein expression of Atg7, Atg5, Beclin, and NCOA4 after 12 h treatment with irisin and/or erastin (β-Actin was used as a loading control). (B) No significant effects on p-

p38/p38, pERK/ERK, and pJNK/JNK levels were found. Erastin effects on the transcription of system X_c^- (*SLC7A11*, *SLC3A2*, *GPX4*), ROS metabolism (*VDAC2*, *VDAC3*, *HO1*, *NQO1*), and iron metabolism (*FTL*, *FTH1*) genes were enhanced significantly in the presence of irisin (20 μM erastin, 100 nM irisin, 12 h. treatment period). All data are means ± SEMs, n ⩾ 3/group; *p<. .5, **p< .1, ****p < .0001 vs. Erastin group (paired t-tests).

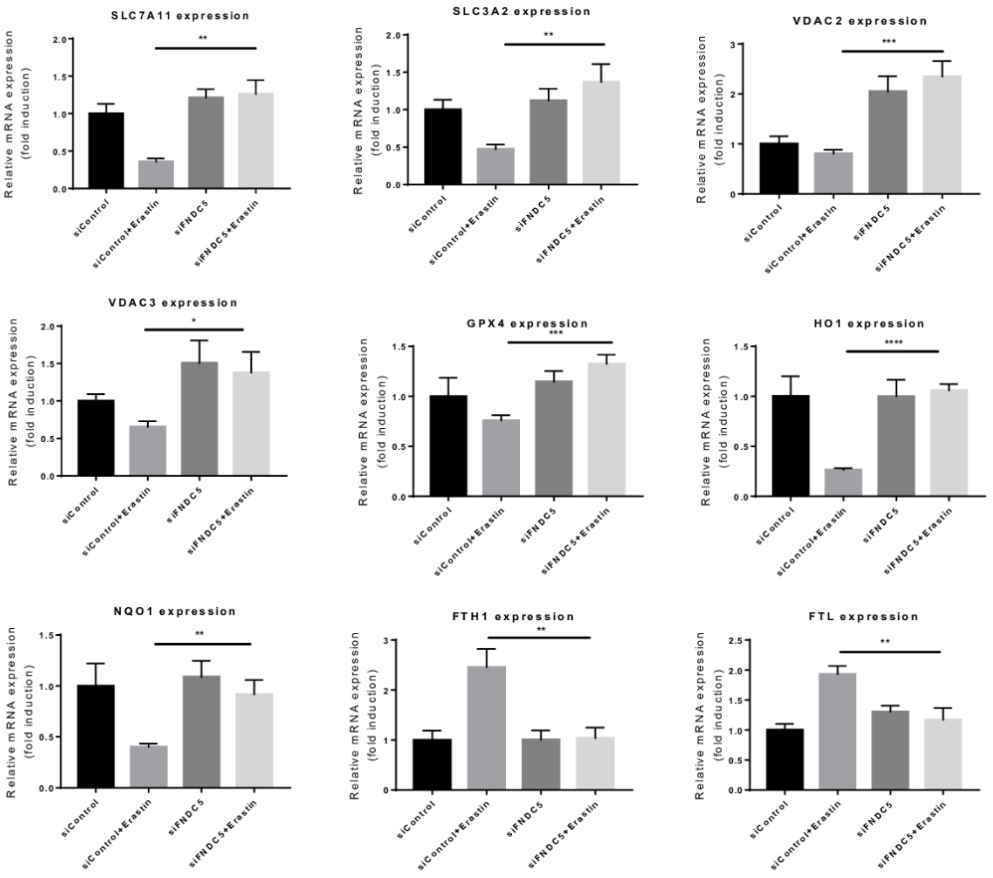

Figure S2. FNDC5 deletion was decreased the erastin-induced ferroptosis in PANC-1 cells. Ferroptosis related mRNA expression **(**system X_c^-, *SLC7A11, SLC3A2, GPX4*; ROS-*VDAC2, VDAC3, HO1, NQO1*; and iron metabolism, *FTL, FTH1***)** were changed after 12 h treatments with 20 μM erastin and/or 100 nM irisin. All data are expressed

as mean ± SEMs, $n \geqslant 3$; **$p < .01$, ***$p< .001$****$p < .0001$ vs. erastin group (paired t-tests).

Table S1. Polymerase chain reaction primer sequences.

Gene	Forward (5'→3')	Reverse (5'→3')
siFNDC5	CCCAAUAACAACAAGGAAATT	UUUCCUUGUUGUUAUUGGGTT
siControl	UUCUCCGAACGUGUCACGU	ACGUGACACGUUCGGAGAA
Human ACTB	TGTCCACCTTCCAGCAGATGT	CGGACTCGTCATACTCCTGCTT
Human SLC7A11	TGGGTGGAACTGCTCGTAAT	AGGATGTAGCGTCCAAATGC
Human SLC3A2	CTCGTGGTTCTCCACTCAGG	CCGCAATCAAGAGCCTGTCT
Human VDAC2	TTGGTTACGAGGGCTGGC	CGAAGTTACTCCTTGTCAGCTTTG
Human VDAC3	CAGACCCTTCGACCAGGAGT	TTCGCAACCCCTAGACTTCAG
Human HO1	CCAGGCAGAGAATGCTGAGTTC	AAGACTGGGCTCTCCTTGTTGC
Human NQO1	CCTGCCATTCTGAAAGGCTGGT	TGATGGAAAGCACTGCCT
Human FTH1	AAGATGGGTGCCCCTGAAG	CCAGGGTGTGCTTGTCAAAGA
Human FTL	CAGCCTGGTCAATTTGTACCT	GCCAATTCGCGGAAGAAGTG
Human GPX4	ACGTGGCCTCGCAATGCGGCAAAA CTGA	TCAGTTTTGCCGCATTGCGAGGCCA CGT

Table S2 Antibodies used in western blotting.

**Antibody**	**Dilution**	**Host species**	**Supplier**
LC3	1:1000	Rabbit	Abcam
NRF2	1:1000	Rabbit	Santa Cruz
p62	1:1000	Rabbit	Cell Signaling
FNDC5	1:1000	Rabbit	Cell Signaling
Atg7	1:1000	Rabbit	Cell Signaling
Atg5	1:1000	Rabbit	Cell Signaling
Beclin1	1:1000	Rabbit	Abcam
NCOA4	1:1000	Mouse	Abcam
p-p38	1:1000	Rabbit	Cell Signaling
p38	1:1000	Rabbit	Cell Signaling
pERK	1:1000	Rabbit	Cell Signaling
ERK	1:1000	Rabbit	Cell Signaling
pJNK	1:1000	Rabbit	Cell Signaling
JNK	1:1000	Rabbit	Cell Signaling
HRP-anti-rabbit IgG	1:1000	Donkey	Amersham
HRP-anti-mouse IgG	1:1000	Sheep	GE Healthcare
